# From voice to ink (Vink): development and assessment of an automated, free-of-charge transcription tool

**DOI:** 10.1186/s13104-024-06749-0

**Published:** 2024-03-29

**Authors:** Hannah Tolle, Maria del Mar Castro, Jonas Wachinger, Agrin Zauyani Putri, Dominic Kempf, Claudia M. Denkinger, Shannon A. McMahon

**Affiliations:** 1 Division of Infectious Diseases and Tropical Medicine, Center of Infectious Diseases, Heidelberg University Hospital, Heidelberg, Germany; 2 Heidelberg Institute of Global Health (HIGH), Heidelberg University Hospital, Heidelberg, Germany; 3 Scientific Software Center, Heidelberg University, Heidelberg, Germany; 4 Johns Hopkins Bloomberg School of Public Health, Baltimore, MD USA; 5 Partner Site Heidelberg University Hospital, German Centre for Infection Research (DZIF), Heidelberg, Germany

**Keywords:** Qualitative research, Interview, Transcription, Speech-to-text algorithm, Automated speech recognition, Whisper, Vink

## Abstract

**Background:**

Verbatim transcription of qualitative audio data is a cornerstone of analytic quality and rigor, yet the time and energy required for such transcription can drain resources, delay analysis, and hinder the timely dissemination of qualitative insights. In recent years, software programs have presented a promising mechanism to accelerate transcription, but the broad application of such programs has been constrained due to expensive licensing or “per-minute” fees, data protection concerns, and limited availability of such programs in many languages. In this article, we outline our process of adapting a free, open-source, speech-to-text algorithm (Whisper by OpenAI) into a usable and accessible tool for qualitative transcription. Our program, which we have dubbed “Vink” for voice to ink, is available under a permissive open-source license (and thus free of cost).

**Results:**

We conducted a proof-of-principle assessment of Vink’s performance in transcribing authentic interview audio data in 14 languages. A majority of pilot-testers evaluated the software performance positively and indicated that they were likely to use the tool in their future research. Our usability assessment indicates that Vink is easy-to-use, and we performed further refinements based on pilot-tester feedback to increase user-friendliness.

**Conclusion:**

With Vink, we hope to contribute to facilitating rigorous qualitative research processes globally by reducing time and costs associated with transcription and by expanding free-of-cost transcription software availability to more languages. With Vink running on standalone computers, data privacy issues arising within many other solutions do not apply.

**Supplementary Information:**

The online version contains supplementary material available at 10.1186/s13104-024-06749-0.

## Introduction

Recent decades have witnessed an ever-increasing use of qualitative approaches in global health research [1, 2], due at least in part to the recognition that in-depth, qualitative insights can add richness to existing data and can facilitate more person-centered, bottom-up solutions to health challenges [3]. However, one factor that limits broader and timelier use of qualitative data is transcription. Transcription refers to the process of converting recorded audio speech, for example from an interview or focus group discussion, into a written format. Transcription is an indispensable part of the qualitative process, and the selection of an adequate transcription approach (e.g. transcribing dialogue versus also capturing utterances such as “uh-huh” or “umm”, details of who is speaking, interruptions, pauses, or involuntary and non-lexical noises such as coughs or throat clearing) is seen as crucial to maintain quality and rigor of data [4, 5]. Nevertheless, the processes and decisions made during transcription represent an often-neglected space within qualitative scholarship, receiving limited attention and reporting in the literature. A recent review about reporting of transcription processes found that 41% of articles employing interviews as a research method did not mention transcription, while 11% mentioned transcripts but not the process of transcription [6]. Given the extensive use of transcription in qualitative research, the limited discourse on the processes, strengths and limitations inherent to transcription is striking [7].

To date, transcription has mainly been accomplished in three ways: by a single researcher or research team who listens to the audio files and manually types text; by professional transcription services wherein recorded material is sent to a company that then returns transcripts; or by software-based transcription programs that entail payment to an external provider, where recorded material is uploaded, automatically transcribed (with or without additional accuracy checks), and transcripts can then be downloaded. Each of these existing approaches entails opportunities and challenges. Manual transcription by the lead researcher or team facilitates extensive engagement with the data, but it is time consuming for the individual(s) transcribing and for the project as a whole. One hour of recorded material typically requires six to seven hours of transcription time [8]. Despite being inherent to the process of manual transcription, delays can lead to collected data waning in relevance [9] or, as witnessed in COVID-19 research [10], becoming obsolete. Many qualitative teams have sought to mitigate transcription delays by forgoing verbatim transcription in favor of selective transcription or via capturing data in the form of field notes and summaries [11, 12]. While selective transcription and related techniques can facilitate timely results, these approaches can increase the risk for researcher bias and information loss [13].

Increasing the number of individuals transcribing a dataset by outsourcing transcription can reduce time but may increase project expenses [14] and cause variability of transcript quality and content, as transcribers may have little familiarity with the research aims [15]. Additionally, in case of emotionally straining research topics or respondent narratives, outsourcing can induce mental stress for transcribers who otherwise would not have come in contact with the data [16]. Data safety and privacy are also a concern when sharing raw data with individuals outside the study team.

Software-based alternatives (e.g., NVivo, TranscribeMe, happyscribe, OneNote (Microsoft) or Smart Pen [17]) are new entrants into the transcription field whose broad utility in academic research has been limited by several factors [18]. In some cases, programs require training on a user’s voice, which is a time-consuming step that reduces the program’s sensitivity to other voices [19]. In other cases, software-based services are expensive and exclusionary, which hinders their use in projects with limited funding or in projects that use languages that transcription firms do not offer within their range of products [20]. Literature on the consistency and accuracy of speech-to-text software is currently limited, but at least one study showed that accuracy varied widely depending on the used algorithm and decreased overall with audio files that were low-quality or entailed multiple speakers [21]. This presents further challenges for researchers since qualitative data often stems from conversational speech (e.g., interviews, focus group discussions wherein multiple speakers and background noise are common). Since software developers often do not provide word-error-rates for this sort of non-naturalized audio recordings, further exploration in this field is necessary [22].

In response to the existing challenges of cost, timeliness, availability, exclusivity and reliability, and with the advent of stronger and less resource-intensive algorithms for everyday use, software engineers and computer scientists worldwide have begun debating feasibility, trade-offs, and opportunities related to transcription via open-source (i.e., free-of-cost) speech-to-text algorithms. Such a platform would mitigate several barriers inherent to manual and/or commercial transcription, but as of now we are not aware of a program that is adjusted to the needs of qualitative researchers, is user-friendly in terms of navigation and is available in an equitable format in terms of language, downloadability and cost.

In this article, we outline our process of developing and adapting a free, open-source, speech-to-text algorithm into a usable and accessible tool for qualitative transcription. We conduct a proof-of-principle assessment of our standalone application, in terms of usability and performance in transcribing non-naturalized audio data in several languages. We further provide a detailed step-by-step guide for researchers considering using this tool for their own data transcription.

## Developing and testing a free transcription software package

### Development

As a first step in developing our transcription tool, we identified available open-source speech-to-text (STT) algorithms including VOSK by Alpha Cephei [23], Silero by SileroAI [24] and Whisper by Open AI [25]. These algorithms were pilot tested using non-naturalized interview data in German in an exploratory approach. We ultimately selected Whisper by OpenAI (see breakout box 1) based on the accuracy and readability of transcripts, the inclusion of punctuation and case sensitive lettering, robustness to background noise, and the program’s potential applicability in numerous languages.

#### Breakout Box 1: Whisper by OpenAI

Whisper by OpenAI is an open-source automatic speech recognition (ASR) system trained on multilingual audio data in an end-to-end approach. OpenAI emphasizes Whisper’s ability to navigate transcription that captures or mitigates challenges related to accents, background noise, and technical language. The algorithm uses one single speech model that automatically recognizes the audio file language and transcribes the data. Audio recordings with mixed languages can therefore also be transcribed easily. Since Whisper was not built via one specific dataset or voice, the system is applicable across qualitative research projects. Furthermore, Whisper runs locally on the user’s computer without requiring a data upload, thereby mitigating privacy concerns. While the program does not require an online connection, running Whisper requires good hardware as it uses between 1–10 GB of RAM, depending on which of the five available speech model sizes is selected. Using Whisper thus entails a trade-off: if a higher level of transcription accuracy is sought, the program’s runtime and RAM requirements will increase.

Like many currently available ASR algorithms, Whisper requires software programming knowledge (e.g. Python) in order to use it for transcribing audio files into text [26], placing it beyond reach for researchers who lack programming skills. Noticing this gap, we developed a standalone application to open the potential of Whisper to a broader pool of researchers. Our goal was to create a downloadable, ready-to-use transcription package that bundles the Python interpreter, the Whisper package, as well as all its dependencies into one standalone tool that allows anyone to run the Whisper algorithm on a personal computer without much effort. We also wanted a product that had an easily navigable user interface and was free to anyone interested in using it for their own research.

The final transcription tool, which we dubbed “Vink” due to its ability of transferring textual data from voice to ink, is available at https://heibox.uni-heidelberg.de/f/6b709d18b0d244cdb792/. More technical information on this standalone application, which was created using PyInstaller, is available online at https://github.com/ssciwr/whisper-standalone/. The tool currently is only available for Windows, the development of macOS and Linux versions is in progress.

When we talk about Vink, we mean our transcription tool which is using the open source STT-algorithm Whisper, and from this point forward we will only talk about Vink unless when explicitly talking about characteristics of the used STT-algorithm.

All assessments were done anonymously and did not include any personal or individually identifiable information. The institutional review board of the medical faculty, University of Heidelberg, Germany, therefore exempted this study from ethical review.

### Proof-of-principle of Vink’s performance on multilingual realistic audio data

We conducted a proof-of-principle assessment of Vink’s performance when transcribing realistic (non-naturalized) audio data in 14 languages including: English (American), Arabic (Classical Arabic), Bahasa Indonesia, Burmese, Chinese (Mandarin), Filipino, French, German, Malagasy, Portuguese (Brazilian), Spanish (Colombian), Tamil, Turkish, and Yoruba.

Multilingual transcription pilot-testers with varying experience in manual audio data transcription each provided one audio file of a discussion in their mother tongue following detailed recording instructions (see Appendix S1). Pilot-testers were selected from the authors’ networks based on interest expressed, languages spoken, and time available. To mimic real-life qualitative data quality, audio files were recorded on either a phone or a regular recording device in a quiet setting. Transcripts of the audio files were generated using the medium size language model of Whisper (5GB RAM required) and were sent back to the pilot-testers for assessment. Pilot-testers were then asked to correct the automatically generated transcript in one sitting, and to record the time needed to correct the transcript and the word error rate (WER) including errors linked to the deletion of filler words (e.g. “uhh” or “umm”); this process facilitated our measure of transcript accuracy. For review instructions, see Appendix S1. Pilot-testers were also asked to complete an anonymous questionnaire on the perceived usefulness of the transcript (see Appendix S2). Following this approach, a total of 19 audio files were provided, 14 of which were assessed. The remaining 5 pilot-testers did not provide an assessment of the transcript (3 contact reminders were sent).

Study data were collected between December 2022 and April 2023, and managed using REDCap electronic data capture tools hosted at the Universitätsklinikum Heidelberg [27, 28].

### Reliability and perceived usefulness of the generated transcripts

Table 1 summarizes the recordings assessed in our proof-of-principle of the algorithm’s transcription performance. Substitutions describe replaced words (e.g. transcribing “house” for “mouse”). Insertions represent added words that were not said, and deletions were cases in which words or non-verbal cues were left out of the transcript.

**Table 1:** Word error rate and time-needed-to-correct of Vink-generated transcripts

Language	Audio length (minutes)	Audio characteristics		Time-needed-to-correct (minutes)		Total words	Word Error Rate (WER)	
American English	06:50	Number of speakers	2	17		854	WER	6.6%
		Sex	F, M				Substitutions	7
		Background noise^1^	Medium				Insertions	50
							Deletions	0
Arabic (Classical Arabic)	03:06	Number of Speakers Sex Background noise	1 F Low	27.5		363	WER Substitutions Insertions Deletions	15.2% 7 20 28
Bahasa Indonesia	05:12	Number of speakers	2	10		465	WER	7.95%
		Sex	F, F				Substitutions	10
		Background noise	Medium				Insertions	22
							Deletions	5
Burmese	05:05	Number of speakers Sex Background noise	3 M, M, F High		Transcript is nonsensical			
Chinese	05:01	Number of speakers	1	12		950	WER	0.95%
		Sex	F				Substitutions	8
		Background noise	Low				Insertions	1
							Deletions	0
Filipino	5:00	Number of speakers	2	19		1343	WER	7.80%
		Sex	F, GNB^2^				Substitutions	56
		Background noise	Medium				Insertions	5
							Deletions	45
French	04:09	Number of speakers	2	19:57		611	WER	24%
		Sex	F, M				Substitutions	15
		Background noise	Medium				Insertions	12
							Deletions	122
German	05:00	Number of speakers	2	9:40		676	WER	4.28%
		Sex	F, F				Substitutions	9
		Background noise	Low				Insertions	2
							Deletions	18
Malagasy	04:41	Number of speakers	2	62		351	WER	41%
		Sex	F, M				Substitutions	134
		Background noise	Medium				Insertions	12
							Deletions	5
Portuguese Brazilian	02:19	Number of speakers	2	4		209	WER	1.4%
		Sex	F, M				Substitutions	2
		Background noise	Medium				Insertions	1
							Deletions	0
Spanish Colombian	06:31	Number of speakers	2	36:46		1111	WER	14.5%
		Sex	F, F				Substitutions	34
		Background noise	Low				Insertions	21
							Deletions	107
Tamil	04:32	Number of speakers	1	72		221	WER	79.8%
		Sex	M				Substitutions	45
		Background noise	Low				Insertions	103
							Deletions	54
Turkish	03:19	Number of speakers	1	8		232	WER	4.3%
		Sex	F				Substitutions	3
		Background noise	Low				Insertions	1
							Deletions	6
Yoruba	5:56	Number of speakers	2	20		528	WER	46%
		Sex	F, M				Substitutions	164
		Background noise	Medium				Insertions	36
							Deletions	45

^1^Background noise levels were classified ‘low’ in case of close to no background noise, ‘medium’ in case of occasional or faint background noises and ‘high’ if background noises notably impaired understandability of speakers ^2^GNB: gender non-binary

The performance of Vink varied widely across languages, with audio files in Chinese, Portuguese, Filipino, English, German, Bahasa and Turkish yielding the most accurate transcripts (WER < 10%), and Malagasy, Tamil and Burmese producing the least accurate transcripts (WER > 40%), according to pilot-testers. As in Radford’s [25] large-scale assessment, the algorithm’s performance did not seem to be language group specific with e.g., high accuracy in Chinese (Mandarin) and extremely low accuracy in Burmese. More likely, this is associated with the very low percentage of e.g. Burmese audio in the training dataset of the Whisper algorithm ( [25]; Appendix E). Among European languages, French required the most extensive transcription correction. The time needed to correct transcripts varied greatly and took between 1.7-fold (Portuguese) and 16-fold (Tamil) the duration of the original audio file.

**Fig. 1:**
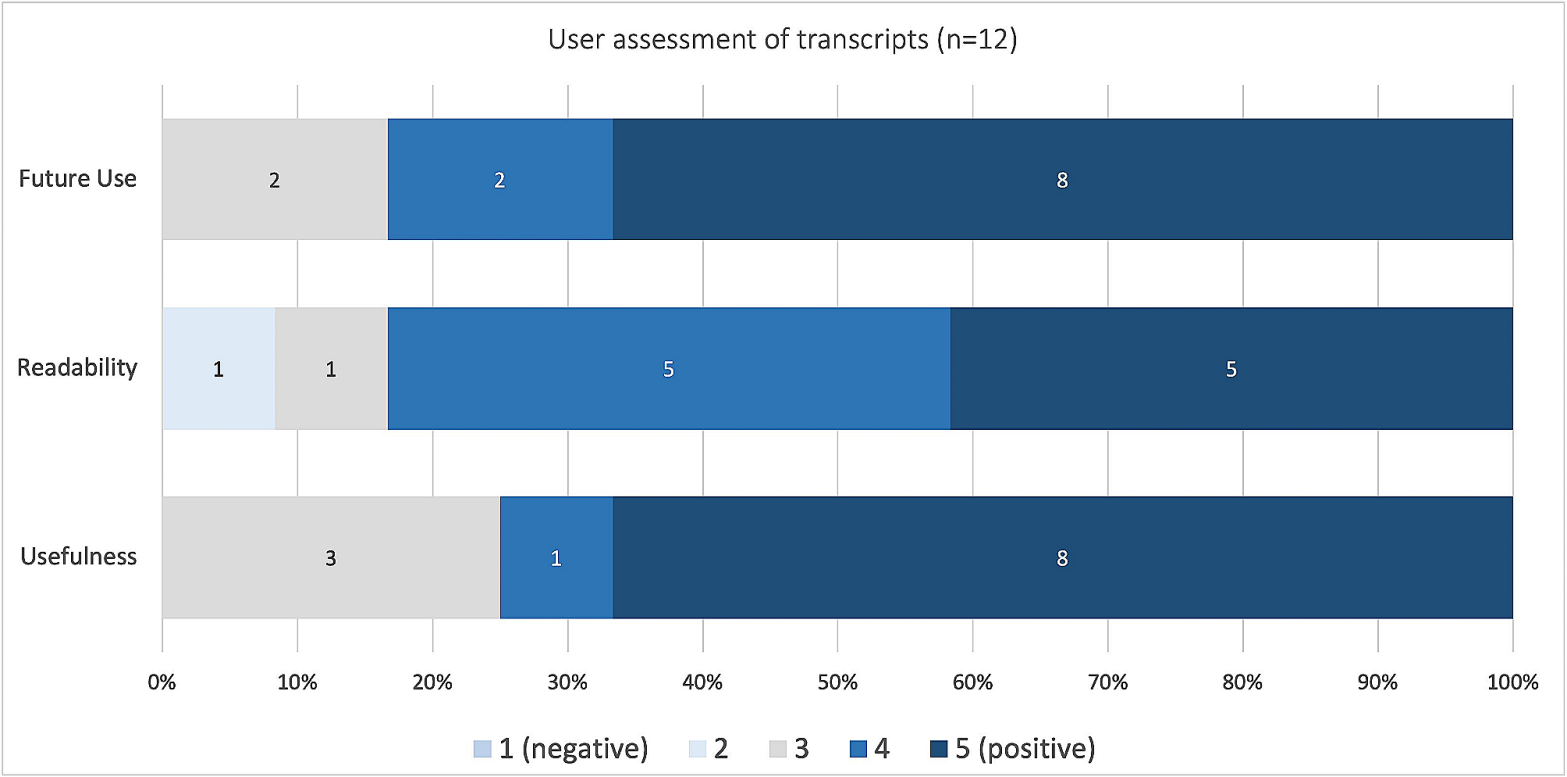
User assessment of generated transcripts– Perceived usefulness, readability of transcripts and likeliness of future use

Overall, most pilot-testers evaluated the generated transcripts positively in the short questionnaire (4 or 5 on a 5-point Likert-scale). The perceived readability of transcripts, which pilot-testers indicated on a 5-point Likert-scale in the short questionnaire, was associated with indication of a low WER category (0–10%, 11–20%, 31–40% or > 40%) of the respective transcript, with an overall high perceived readability across languages. All pilot-testers whose transcript had a WER below 20% (*n* = 9), and a total of 10 out of 12 pilot-testers who completed the short questionnaire, indicated that they were either likely or very likely to use Vink-based automated transcription in their future research. Results of the short questionnaire are presented in Fig. 1.

However, the results from the questionnaire revealed several areas for improvement. First, the algorithm seems to naturalize the text output and therefore rarely includes filler words in the transcript. Non-verbal vocalizations such as laughing, crying or hesitations are omitted as well. Repetitions are partly cleared in the final transcript, producing a denaturalized transcript version [29]. These deleted, non-verbal vocalizations account for a significant part of the WER in our assessment. For instance, the algorithm would naturalize the sentence “We, ehm, wanted to gi-… give an example.” to “We wanted to give an example.”, which would be counted as two deletions in our assessment. Respondents wished for hesitations and pauses to be included and captured with an ellipsis symbol (“…”) rather than a comma.

According to respondents, the algorithm (as described in previous papers on ASR [21, 30]) struggled during crosstalk segments of the audio data. Some respondents suggested that highlighting longer pauses or the different speakers in the audio recording could be helpful, for instance line breaks between speakers. Speaker recognition was also deemed to potentially be helpful to distinguish the different voices, especially if Vink were to be applied for transcribing focus group discussions.

### Usability of Vink

#### Testing the usability of our transcription package and user interface

To gauge the usability of the downloadable package and interface of Vink, we gave 5 people [31] without previous experience in computational science access to the transcription package and provided them with an instruction sheet (see Appendix S3) on how to download and use the transcription tool. We then observed how well users were able to navigate our transcription tool using cognitive think-out-loud interviewing during first use. In addition, we asked users for feedback regarding how they perceived the tool in terms of usability and user-friendliness, and what changes they suggested to increase usability.

### Challenges and improvements

Our usability assessment showed that users were able to independently install Vink and transcribe an audio file using the incorporated interface. Reported issues included difficulties finding the executable file for Vink in the downloaded folder and confusion about suitable text file formats, which were addressed in the latest version of Vink to enhance user friendliness. Inter alia an installer was added to facilitate the set-up process. Most struggles and uncertainties resulted from pilot-testers overlooking content in the instruction manual, highlighting the importance for our team to maximize the self-explanatory nature of the interface. See Table 2 for the complete list of reported usability issues and subsequent improvements.

**Table 2:** Reported usability issues of Vink and changes made

Reported usability issues	Type of change	Description of changes made
Not enough memory space to download Vink Slow download process	Instructions	Required memory space and download time was clarified in the installation process
Not enough RAM for larger models due to too many programs running in parallel	Instructions	Included advice to close other programs that are running in parallel on the PC/Laptop
Search for the.exe file in the downloaded folder to open the application	Modification of the tool	We added an installer to the application
Computer warning about first execution of application	Modification of the tool	Purchase of a code signing certificate; no more need for a firewall exception
A pop up of Windows system window appears before the interface	Instructions	Explanation added to instructions
Confusion about accepted audio file formats	Instructions	The need of an audio file and required formats are described more prominently
Confusion about required text output file and format	Modification of the tool	Application outputs generated text into the interface
Confusion about differences between language models	Instructions	Trade-offs between models are explained in more detail
Confusion about choice between CPU and Graphic Card	Instructions	Benefits of using each option are now explained
Confusion about whether the app works offline	Instructions	It is highlighted that the app needs an internet connection to load models

Vink’s interface and the instructions for use were also further modified following a rapid, iterative approach drawing on human-centered design principles. The user manual of the newest version of Vink can be found in Appendix S4.

### Summative evaluation of Vink

Taken as a whole, existing standards for transcription present challenges that can be addressed by ASR algorithms such as Whisper, which can be made accessible via standalone applications such as Vink. Table 3 summarizes overarching challenges to traditional verbatim transcription, how Whisper as an ASR algorithm can address some of these challenges, how Vink influences the usability of Whisper for audio transcription, and what additional needs persist.

**Table 3:** Needs of traditional transcription, opportunities via whisper and additional opportunities via Vink

Transcription concerns and needs	Characteristics of Whisper	Characteristics of Vink and additional needs
**Resources, infrastructure, and costs**		
Transcription services are expensive.	Whisper is offered by OpenAI free of cost.	Vink is a free of cost transcription tool using Whisper’s open-source algorithm.
Transcription software often requires high computing power to operate.	Whisper offers multiple model sizes that require 1–10 GB of RAM, thus can run on average computers, depending on the model size.	Vink conserves this feature from Whisper, allowing selection of model size per user and computer characteristics.
**Safety and privacy**		
Uploading data for transcription or outsourcing transcripts to a third party raises confidentiality and data protection issues.	Whisper runs locally, thus eliminates the need to share or upload data.	Vink is designed to operate locally without uploading data.
**Quality of transcription**		
Transcription software is often unavailable in non-Western or less dominant languages.	The same speech models for all languages technically make Whisper usable for everyone, yet differences in performance persist. Audio files with mixed languages can be transcribed.	Accuracy of transcription varies across languages (Table 1).
Conventional transcription software often requires training on a user’s voice or on exemplary audio data.	The Whisper algorithm has already been trained on big data and is ready for use.	The ‘ready to use’ feature limits the possibilities to adapt the algorithm to individual requirements.
Conventional transcription software often struggles with accents, mixed use of languages and background noise.	Whisper provides improved robustness to accents, background noise and technical language.	The improved speech recognition comes at the expense of expressions (e.g., laughter) that are excluded from the final transcript.
Identifying speakers (e.g., interviewer, respondent, multiple participants) is an essential but sometimes challenging feature of transcription.	Whisper does not offer speaker recognition.	Vink currently does not include speaker recognition. Depending on the transcription approach, the user may need to add them manually.
Other open-source transcription software (Silero, Vosk) only output raw lower-case text. Punctuation models can be applied later in the process, but these are not available for all languages.	Whisper generates transcripts with already integrated punctuation and upper cases regardless of the language.	
**Ease of use**		
Transcription software should be accessible to researchers without knowledge of software programming.	Whispers requires a programming language (e.g., Python, R), an interpreter and installation of specific packages within the programming software, to operate.	Vink is a downloadable standalone application which includes the necessary packages and tokenizers, reducing the installation requirements and steps.
	Whisper does not have a user interface, which limits its use to people with knowledge of programming (e.g., Python).	Our transcription tool includes an intuitive user interface.

## Discussion

Vink is an easy-to-use, open-source speech-to-text tool that facilitates the use of the Whisper ASR-algorithm for non-programmers in qualitative research. It is free of cost, making it an accessible transcription solution for research projects. The usability for transcribing audio files in non-western and (in a research sense) rarer languages, as well as the limited computing power required to operate it, make our transcription tool usable for everyone with access to a standard computer or laptop. These characteristics may help mitigate global disparities in health research resources [32]. In addition, compared to uploading data to third-party transcription services, Vink runs locally, which allows protection of privacy and confidentiality of data, an established principle of qualitative research [33, 34].

The accuracy of generated transcripts is central to the application’s value in qualitative research. Poland [35] defined transcription accuracy as faithfulness to the original speaker’s intention and fit with the research aims. In practice, transcripts are often considered accurate when they match the recorded audio, disregarding the original interaction. Although problematic as this takes a purely positivist view that there is one ‘correct’ version, this understanding allows for a comparison of transcripts and presents a feasible common ground for accuracy assessment in our case. Part of this consideration on transcript accuracy is the inclusion of behavioral annotations. Gestures and non-verbal vocalizations can be considered representative of e.g., the speakers’ engagement in the interview or topic, or their certainty in their expressed opinions. However, non-verbal cues are often excluded from transcripts, whether transcribed by hand or with algorithm support. This form of ‘selective transcription’ increases readability but loses data and risks researcher bias. By virtue of saving time on the pure documentation of words, Vink may allow researchers to invest more time in capturing and annotating the broader context of the interview or focus group discussion.

In Radford’s [25] large scale and our proof-of-principle assessment of Whisper’s accuracy on multilingual speech, the overall performance (or word-error-rate (WER)) of the algorithm is good. Variability in WERs show that despite the algorithm technically being applicable to a high number of languages, remarkable disparities in accuracy remain across languages, commonly favoring languages such as English, German, and Chinese. In a few languages that are linguistically more distant from English, or for which the amount of audio data used in training Whisper was comparatively low ( [25] Appendix E), the quality and therefore usefulness of the transcripts decreased. While the amount of respective audio data for training is strongly correlated with Whisper’s performance, an additional factor for those languages is a lack of transfer due to the linguistic distance from English, which was predominantly (65%) used for training Whisper.

The lack of transparency regarding metrics in machine learning literature [36], including the exact definition of the WER in the original publication on the Whisper algorithm [25], challenges comparisons across programs. For example, it is not clear whether filler words are considered in the WER assessment. Such deletions are relevant for qualitative research, as pauses for example can indicate divided attention or nervousness of the interviewee [37], and most word errors in our assessments were due to deletions of non-verbal vocalizations. However, the WER as a metric does not account for the causes of errors. Factors that can affect WER, independent of the capabilities of the ASR technology, include recording quality, technical terms or proper nouns, background noise, sex of the speaker, pronunciation, and speech fluency. These factors might explain the differences in WER between our own assessment and the large scale original assessment of Whisper’s WER [25]. With the limitations of the WER, other parameters (e.g., perceived usefulness or time-needed-to correct) provide valuable information for a realistic assessment of the transcript’s value for researchers. In our findings, the readability of transcripts was generally perceived as high, which implies an accelerated process of correction since the text can be followed and adjusted more easily. However, our preliminary assessment can only provide first insights into practical performance of Vink in real-life research scenarios; we would encourage scholars employing Whisper or Vink in their work to share their own experiences or further large-scale assessments.

Researchers have argued that computers may tempt qualitative scholars to perform ‘quick and dirty’ research [38] and could lead to a loss of closeness to the data [39]. In the context of automated transcription, we see the risk of generated text being superficially evaluated in terms of its readability and not by its nuanced representation of the original recording, including non-verbal cues. Additionally, the Whisper algorithm is trained to condition on the history of text of the transcript in order to use longer-range context to resolve ambiguous audio [25]. Sentences with non-understandable parts are reconstructed leading to overall higher accuracy and good readability but possibly a false sense of certainty of transcript correctness in hard-to-understand passages. We therefore advocate for researchers considering using speech-to-text tools (including Vink) to carefully choose its exact mode of application. Especially for researchers interested in nuances of human interaction, too much reliance on the automatically generated transcript might cause a significant loss of valuable data. The applicability of automated transcription is also challenged by scholars such as Lapadat [40] who view transcription as a process rather than a product, as it involves constant decisions regarding how to present the data and which additional information to include. This makes transcription an inherently interpretative act, influenced by the transcriber’s own biases and assumptions [41]. As algorithms are not able to make such decisions about meaning-making and interpretations, nor about ways in which these meanings may best be represented [21], we propose that ASR generated transcripts should merely be seen as a first step in the transcription process, and are to be revised and modified [42].

In terms of limitations, Vink as of now is only available for Windows computers, which restricts its potential user base. We are currently working on macOS and Linux versions. Additionally, our assessment of the time-needed-to-correct and WERs across languages was designed for proof-of-principle purposes. Despite efforts to provide as detailed descriptions for transcript correction and assessment as possible, pilot-testers’ varying levels of experience in transcribing or correcting qualitative data may have introduced variation in the time-needed-to-correct and WER assessments. Larger, systematic evaluations of the algorithm’s performance, building on the assessment by Radford et al. [25], and evaluations of Vink’s usefulness in facilitating qualitative research transcription processes would provide additional insights. Similarly, we did not assess the algorithm in several contexts relevant for qualitative research (e.g., focus group discussions, speech with strong accents, more background noises). As qualitative research often is performed in settings where the researcher only has limited control over environmental factors, such further assessment would allow a firmer establishment of the conditions required for the algorithm performance to be sufficiently useful in the particular context of qualitative research.

### Going forward

A step-by-step guide on how to install and use Vink is available for use (Appendix S4). The code for the graphical user interface of Vink, as well as the combined work with the bundled dependencies are published under the MIT license. Vink’s installer will also install a number of bundled software packages under a variety of software licenses (Nvidia License Agreement for Nvidia SDKs, LLGPL v3, MPL v2, PSF License, Apache 2.0, BSD-3, BSD-2, MIT, Zlib license, Unlicense). For detailed information about these licenses, please read the license agreement. We ask users to credit OpenAI when using the algorithm, and to cite this publication when using Vink in their own work. As mentioned, Vink-generated transcripts should be seen as a first step in the transcription process, which are to be revised by research teams (and ideally, those who undertook the data collection activity and/or who will undertake data analysis).

We are happy to hear about other researchers’ experiences, successes, and challenges in applying this approach to automatic transcription in their own work and are open to feedback and suggestions. We intend to make a portal for feedback available, in the meantime please contact the corresponding author. Additional guidance and information on the Whisper algorithm are available online (not moderated by us), for example at https://openai.com/research/whisper or https://github.com/openai/whisper. Tutorials and forums to chat about possibilities and limitations of automated speech-to-text transcription are emerging, allowing for an exchange between interested individuals. To the best of our knowledge, such forums are primarily technical in nature.

We aim to improve and update the standalone package in the future. Improvements in language models, when published by Open AI, will be considered in newer versions. Being based on an open-source algorithm means that the way this program operates is more transparent than in commercial software and can be examined by the research community.

## Conclusion

In this article, we have introduced and evaluated our novel transcription tool Vink for automated interview transcription in various languages, based on OpenAI’s Whisper. Our findings outline the possibilities of integrating open-source speech-to-text algorithms into qualitative research. With the current rapid developments in this field, we expect the accuracy, relevance, and ease of use of ASR to continue to increase, and we want to contribute to the emerging discourse on its resulting potentials and drawbacks for qualitative research. We hope that by providing a ready-to-use and free tool we will allow qualitative researchers, especially those with limited resources, to save time and money. These resources in turn can be reinvested in engaging more profoundly with data and in deepening other steps of the analytic process, thereby ultimately strengthening the quality of qualitative research across settings and disciplines.

### Electronic supplementary material

Below is the link to the electronic supplementary material.

Supplementary Material 1

Supplementary Material 2

Supplementary Material 3

Supplementary Material 4

## Data Availability

No datasets were generated or analysed during the current study.

## Declarations

All assessment for perceived usefulness was done anonymously and did not include any personal or individually identifiable information.

The assessment for usability of the application was done within the research group.

The institutional review board of the medical faculty, University of Heidelberg, Germany, which was consulted when planning this project, therefore exempted this study from ethical review.

## Abbreviations

ASR: Automated Speech Recognition
F: Female
GB: Gigabyte
GNB: Gender non-binary
M: Male
RAM: Random-Access Memory
STT: Speech-To-Text
WER: Word Error Rate
