# Publisher Correction: From voice to ink (Vink): development and assessment of an automated, free-of-charge transcription tool

**DOI:** 10.1186/s13104-024-06842-4

**Published:** 2024-07-18

**Authors:** Hannah Tolle, Maria del Mar Castro, Jonas Wachinger, Agrin Zauyani Putri, Dominic Kempf, Claudia M. Denkinger, Shannon A. McMahon

**Affiliations:** 1 Division of Infectious Diseases and Tropical Medicine, Center of Infectious Diseases, Heidelberg University Hospital, Heidelberg, Germany; 2 Heidelberg Institute of Global Health (HIGH), Heidelberg University Hospital, Heidelberg, Germany; 3 Scientific Software Center, Heidelberg University, Heidelberg, Germany; 4 Johns Hopkins Bloomberg School of Public Health, Baltimore, MD USA; 5 Partner Site Heidelberg University Hospital, German Centre for Infection Research (DZIF), Heidelberg, Germany

**Publisher Correction: BMC Research Notes (2024) 17:95**

10.1186/s13104-024-06749-0

Unfortunately, some of the author’s feedback on the article proof was inadvertently published online.

The incorrect text has been removed from the affected sections of the article - final paragraph of the introduction, header of Table 1 and header of Table 3.

The original article has been corrected.

