# Supplementary material for: From voice to ink (Vink): development and assessment of an automated, free-of-charge transcription tool
Source: BMC Res Notes. 2024 Mar 29;17:95. doi: 10.1186/s13104-024-06749-0 (PMC10981346; doi:10.1186/s13104-024-06749-0)
Supplement: Supplementary file 1 — Supplementary Material 1 [file 13104_2024_6749_MOESM1_ESM.docx]

**Proof-of-principle assessment instructions**


**Transcription Software Evaluation**

Thank you again for signaling potential interest in contributing audio recordings to support the evaluation of a novel, free transcription approach. As discussed, we would like to give you some more information on what this evaluation is about and what your participation would entail.

**What is the aim of this project?**

We would like to assess the performance of a new, open-source transcription software (whisper) in transcribing authentic audio data in different languages. The successful use of free transcription software could make a big difference towards making qualitative analysis less resource and time intensive, especially in a global health perspective.

**Which languages are we interested in?**

In our first evaluation we would like to focus on some rather common languages that are of interest in global health research. Therefore, we are searching for volunteers who speak ***English, French, Spanish, Portuguese, Swahili, Filipino, Arabic or Chinese***. However, we are also interested in expanding this list, depending on the availability of audio recordings– please reach out to [name of the responsible co-author] (see below) if you would like to offer another language.

**What would your contribution entail?**

We would kindly ask you to ***record a discussion with a friend or family member in your native language***. This audio recording should ideally be about 3-5 minutes long. If it is possible, we would prefer having recordings with a male and female voice.

To ensure realistic quality of the audio data we would prefer it if you could record your discussion on either a phone or a regular recording device in a rather quiet setting without distractions or too much background noise. Please don’t eat or drink while making the recording. However, if you absolutely cannot avoid having some noise on your recording this is fine as well.

We would ask you to talk as freely as possible. Please don’t try to speak extra clearly, since we are particularly interested in authentic real-life audio data. ***We want to highlight that we will not analyze any content of the shared audio files, nor will the transcript be shared with anyone besides the person running the software and the person evaluating it (ideally you, see below) without your explicit prior approval. Transcripts and audio files will be destroyed immediately after evaluation is complete***. Please do not be overly conscious of what exactly you discuss – it can be as mundane as you want, as long as it has a similar flow to a normal conversation.

**Ideas for discussion topics:**

However, to make the discussions easier for you and to ensure a comparability of the language level of the different recordings, we would suggest you talk about one or several of the following topics:

- What does global health mean to you?
- How does your work relate to global health?
- Which experiences have you made with qualitative data collection?
- Which global health fields are you most interested in and why?

Once you have finished your recording, ***please submit it to [responsible co-author and address].***

**What comes after recording and how does the evaluation work?**

Once we have received your recording, we will run the transcriptions software on your audio data. To ensure maximum accuracy and privacy, it would be great if you would also be available to assess the transcription’s accuracy of your own recording (we expect this to not take more than 15 minutes of your time). If you are available for this, we would then send the resulting transcript of your recording back to you (most likely within a week of your submission).

We would then ask you to ***please assess how well the automatic transcription performed on your audio data***. We would like you to do this by correcting the transcript (by relistening your recording) and determining the word error rate of the transcription (how many words did the program get wrong in relation to how many words there are in total), as well as an estimation of how long it took you to correct the transcript. However, we would share a more detailed description on the evaluation criteria when sending you the transcript. To conclude we would then ask you to answer 3-4 short questions on how useful you perceived the transcription.

We would be very thankful if you decided to support this project. If you have any questions, please don’t hesitate to reach out to [responsible co-author] (for organizational questions, [e-mail address]) or [first author] (for software/technical questions, [e-mail address]).

**Instructions for assessment of the generated transcripts**

Dear Participant,

Thank you very much for having contributed an audio file to our project.

You will find attached the transcript of your audio file which was generated automatically using our transcription application based on Open AI’s “Whisper”.

As mentioned before, we would like you to assess the transcription’s accuracy of your own recording to ensure maximum accuracy and privacy (we expect this to not take more than 15 minutes of your time). Therefore, ***we would like to ask you to correct the transcript that was sent to you.***

Please see below some specific instructions regarding how to assess the transcript accuracy. If you have any questions, please reach out to me [e-mail address].

- Please correct the transcript while relistening to your original audio recording and **measure the time you needed to correct the transcript**. To ensure that the time measured reflects the actual time needed to correct, please correct the transcript in one single sitting. Please feel free to use the transcription approach that you feel most comfortable with and that you would use when transcribing by hand yourself.
- **Word error rate** (this will allow us to make comparisons with other software, open and paid).

Please count how many words the transcription software “got wrong”, how many it added and how many are missing. For detailed description of how to calculate the WER, please see the explanation below. Please also consider filler words.

**Please enter the results of your assessment (WER and time needed to correct) in the table on top of the transcript and send the corrected version to [name and address of responsible co-author].**

To conclude we would then ask you to answer four short questions on how useful you perceived the automatically generated transcript. For this, we have created a fully anonymous questionnaire on RedCap which you can access via the following link:

[Link to anonymous short questionnaire]

**Thank you very much for your time and effort!**

**How to calculate the word error rate (WER)?**

The WER is the number of errors divided by the total words, according to the formula:

**Word Error Rate = (Substitutions + Insertions + Deletions) / Number of Words Spoken**

To calculate the WER, please start by adding up the substitutions, insertions, and deletions that occur in a sequence of words in the transcript, as described below:

- A **substitution** occurs when a word gets replaced (for example, “noose” is transcribed as “moose”)
- An **insertion** is when a word is added that wasn’t said (for example, “SAT” becomes “essay tea”)
- A **deletion** happens when a word is left out of the transcript completely (for example, “turn it around” becomes “turn around”)

Then, divide that number by the total number of words originally spoken. The result is the WER.

This can be illustrated with an example^[[1]](#footnote-1)^:

Let’s say that a person speaks 29 total words in an original transcription file. Among those words spoken, the transcription included 11 substitutions, insertions, and deletions.


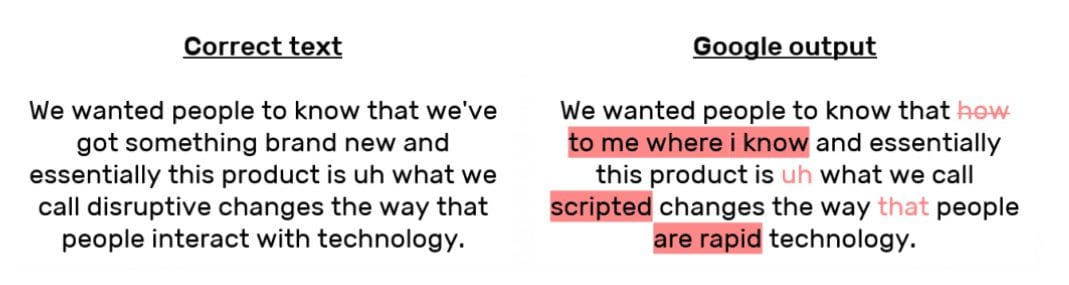


To get the WER for that transcription, you would divide 11 by 29 to get 0.379. That rounds up to .38, making the WER 38 percent.

Taken from: https://www.rev.com/blog/resources/what-is-wer-what-does-word-error-rate-mean#:~:text=Basically%2C%20WER%20is%20the%20number,The%20result%20is%20the%20WER.

1. [↑](#footnote-ref-1)
